# Evaluation of the parents’ anxiety levels before and after the diagnosis of their child with a rare genetic disease: the necessity of psychological support

**DOI:** 10.1186/s13023-021-02046-2

**Published:** 2021-09-28

**Authors:** Ayse B. Kolemen, Enes Akyuz, Ali Toprak, Erdem Deveci, Gozde Yesil

**Affiliations:** 1 Department of Medicine, Bezmialem Vakıf University Faculty of Medicine, Istanbul, Turkey; 2 Department of Biophysics, Saglık Bilimleri University Faculty of International Medicine, Istanbul, Turkey; 3 Department of Biostatistics and Medicine Informatics, Division of Basic Medical Sciences, Bezmialem Vakıf University Faculty of Medicine, Istanbul, Turkey; 4 Department of Psychiatry, Medipol University Faculty of Medicine, Istanbul, Turkey; 5 Department of of Medical Genetics, Istanbul University Faculty of Medicine, Istanbul, Turkey

**Keywords:** Rare genetic disease, Anxiety, STAI, Parents, Psychological support

## Abstract

**Background:**

The diagnosis of the rare genetic diseases has great importance in treating multisystemic conditions, preventing potential complications, and estimating disease risk for family members. The duration of obtaining genetic test results is varies. The demand to learn the diagnosis of a possible untreatable illness involves a struggle between uncertainty and a lifetime chronic disease. The current uncertainty of their child's condition and the long wait for a diagnosis may increase the parents' anxiety level and cause difficulties in the continuation of diagnostic procedures in some families. This study aimed to investigate the prediagnosis and postdiagnosis anxiety levels of parents who have a child with a rare genetic disease.

**Method:**

The parents in this study, mothers or fathers, admitted their children to the Bezmialem Vakıf University Medical Genetics Clinic due to a suspected rare genetic disease (n = 40). Researchers created “The Sociodemographic Questionnaire” and used it to analyze the parents' sociodemographic status. In addition, they used the State-Trait Anxiety Inventory (STAI) to determine the anxiety levels of the parents.

**Results:**

The state anxiety levels of parents decreased significantly after learning the diagnosis. However, there was no statistically significant decrease observed in trait anxiety levels.

**Conclusion:**

Data from this study revealed that informing parents about their child's disease and properly explaining to them the expected difficulties might help to reduce their anxiety levels. Psychological support for parents is necessary to reduce their long-term stress, thus increasing the patient's compliance with treatment.

**Supplementary Information:**

The online version contains supplementary material available at 10.1186/s13023-021-02046-2.

## Background

There are approximately 7,000 different types of rare diseases defined thus far. The definition of a rare disease may differ among countries. For instance, it is defined as a condition that affects fewer than 1 in 1500 people in the United States, 1 in 2500 individuals in Japan, and 1 in 2000 people in Europe. Approximately 350 million individuals have been affected by a rare disease globally, and 75 percent of them are children [1–3]. The onset of rare genetic diseases can occur at birth or during infancy. Approximately 80% of rare diseases have genetic origins. Most rare genetic diseases are incurable and affect many body systems throughout life. A prolonged process to diagnose the rare disease may worsen the situation. According to studies, 30% of individuals with a rare disease die before age 5 [3, 4].

Meeting the needs of a child with a rare genetic disease takes much more time than raising a healthy child. When parents have a child with a rare genetic disease, new roles and responsibilities are assumed, which can be a significant source of stress for the parents. They may struggle to adapt to new roles and responsibilities [5, 6].

Insufficient knowledge of rare disease challenges physicians when diagnosing their patients. Despite modern-day advanced molecular genetic diagnostic systems, it is not possible to diagnose all rare genetic diseases [2, 7]. Waiting to be diagnosed could be challenging for patients and their parents. Most such patients visit multiple physicians, and many years may pass before they obtain a correct diagnosis. According to the survey, the average duration of diagnosing a rare disease is 5.6 years in the United Kingdom and 7.6 years in the United States. Parents may feel lonely and hopeless during the diagnostic process because rare diseases are not often seen, and there is not much information about them. [8–12]. During these stressful times, parents can struggle to find time for their relationship, which may damage their marriage [13].

The diagnostic process of the rare disease takes time as well as its care could be difficult and very costly. Additional hospital care and visits and the price of the orphan drugs can be expensive. Especially, children in underdeveloped and developing countries might not get the necessary care. The worldwide policy and action plan are still needed to give better healthcare to children with rare diseases [6, 14, 15]

A broad understanding of the etiology of anxiety is that it includes several factors, such as biological, psychological, and social determinants, mediated by various risk and protective factors. Variables associated with distress in the parents of a child with a rare genetic disease may include the severity of the illness, low family income, being a mother, working, and a long period without a diagnosis [16–18].

Mothers are the primary caregivers of the children in the vast majority of cases. Therefore, they could have more responsibilities than the father or other caregivers. They can find themself in a stressful position and under pressure. Many studies have concentrated on determining the consequences and factors connected to parenting stress in mothers [19]. In addition, mothers are more psychologically affected than fathers by their child's health condition. The study showed that despite intensive treatment of a sick child, parents' anxiety levels could be high, especially in mothers. Additionally, parental anxiety levels might not change during the treatment process [20]. In line with the literature, another study found that mothers of children with rare diseases had higher levels of anxiety than fathers [18].

Parents of children with chronic illnesses are more likely to suffer from depression and anxiety-related illnesses. They can use different strategies to cope with that distress. Adaptive coping strategies, such as talking with friends and family, exercising, or praying, may improve general well-being. Based on disease severity, parents can experience grief over their child's health. Grief is a natural process when reacting and coping with a loss. The stages of grief consist of denial, anger, bargaining, depression, and acceptance. Individuals do not have to experience all stages while grieving, and there is no order among the stages. Everybody has a special way to cope with sorrow. The parents of a child with a rare genetic disease might have grief-related feelings over their child's health. This grief can sometimes cycle and does not resolve completely. It may restart when new symptoms or issues arise [21–25].

It was emphasized in the studies that psychological support is required for the family of a chronically ill child because of the high stress, depression, and anxiety levels of parents. Cognitive-behavioral and existential psychotherapy are common methods to treat the emotional distress of children and their parents [26–28]. Previous studies have shown that helping parents cope with stress will contribute into the treatment process of sick children. Symptoms of a chronically ill child can improve by reducing parental distress. Additionally, the low stress level of the parents can protect the child from sorrow and anxiety [28–30].

We expected to see that anxiety levels would be high in the parents of a child with a rare genetic disease. Therefore, this study aimed to show the necessity of psychological support for parents who have a child with a rare genetic disease. We believe that psychological support helps parents cope with pre- and postdiagnostic stress and helps them better care for their child. Additionally, in this study, we investigated parental sociodemographic status and its effects on anxiety levels.

## Method

### Aim

This study investigates the evaluation of pre- and postdiagnostic anxiety levels of parents who have a child with a rare disease and investigates parental sociodemographic status effects on their anxiety levels.

### Participants

The study sample consisted of 40 randomly selected parents, mothers or fathers, of a child who applied to the Bezmialem Vakıf University Medical Genetics Clinic in 2017–2018 with a suspected rare genetic disease. The family decided which parent would join the interview. Both parents of the child joined the interview in 6 families. An additional table shows the type of rare disease of our patients (see Additional file 1: Table S1).

### Design

Two interviews were conducted in this study to evaluate parental anxiety levels, one before and one after they received their child's diagnosis. Interviewers used the Form X version of the State-Trait Anxiety Inventory (STAI) to measure parental anxiety levels [31]. Each parent was called the day before their appointment. The call reminded them of the appointment day and asked whether they would participate in the survey. The survey was administered to parents who were willing to participate. If the parents were not available when they received the call, they participated in the test face-to-face immediately before learning the diagnosis. The second interview was conducted in the two weeks after the diagnosis. The second interviews were provided face-to-face or by phone according to the availability of the parents. Researchers presented the created sociodemographic information form to the parents when they first came to the clinic. They used the questionnaire to evaluate parents’ sociodemographic status effects on their anxiety levels. This study was approved by Bezmialem Vakıf University's ethics committee (approval number: 10/15).

### Data collection

#### Personal demographic ınformation form

The researcher created the questionnaire, consisting of 8 questions, to obtain sociodemographic information about the family. The parents were asked about their parental status, age, occupation, educational status, income level, marital status, number of children, and psychiatric pharmacological treatment.

#### The Spielberger's trait-state anxiety ınventory

The State-Trait Anxiety Inventory (STAI) is defined as a self-evaluation questionnaire for measuring anxiety levels in individuals. Form X of the STAI includes two scales, designed to evaluate the transient state of state anxiety and the long-standing quality of trait anxiety. The state anxiety scale (the S-Anxiety scale) determines how individuals feel at a particular moment in certain conditions. The trait anxiety scale (the T-Anxiety scale), on the other hand, determines how an individual feels in general, regardless of his/her circumstances. The state and trait anxiety scales together consist of 40 multiple-choice items, and 36–41 is an average score. Each scale has 20 expressions that are evaluated as 4-point Likert items ranging from 1 (not at all) to 4 (very much) for the status scale reflecting intensity [31–33].

### Statistical analysis

The data were analyzed using the IBM Statistical Package for Social Sciences (SPSS) 22.0 statistical program [34]. The Shapiro–Wilk test was used to check the distribution of the data. For each anxiety scale, the average of the scores obtained from the individuals was calculated. The differences in the continuous dependent variables within the group were analyzed by the Wilcoxon Sign Test. The sociodemographic data were analyzed. For each group, the difference in continuous variables between the groups was examined with the Mann–Whitney U test. The median (min–max), mean ± SD, frequency, and percentage value are given as descriptive statistics. *p* < 0.05 was considered statistically significant.

## Results

The average anxiety level for both the state and trait anxiety tests was 36–41. Before learning the diagnosis, 50% of the parents (n = 20) had an above average state anxiety level, and after they learned the diagnosis, this number dropped to 37% (n = 15); 57.5% of the parents (n = 23) had above average trait anxiety, and this number dropped to 40% (n = 16) after they learned the diagnosis. An additional table shows this in more detail (see Additional file 1: Table S2).

When the scores of the state anxiety scale before and after diagnosis were compared on an individual basis, 60% of the parents (n = 24) had state anxiety scores that were lower than the first ones, and 32.5% (n = 13) had higher state anxiety scores. However, 7.5% (n = 3) of parents had the same state anxiety score. According to the Wilcoxon Sign Test, the state anxiety levels of the parents were found to be significantly decreased after the diagnosis of their child (p = 0.049).

Based on the postdiagnosis trait anxiety scale results, 45% (n = 18) of the parents had a lower trait anxiety score, 35% (n = 14) of the parents had a higher trait anxiety score, and 20% (n = 8) of them had the same trait anxiety score. There was no statistically significant decrease observed in the scores of the trait anxiety scale after the diagnosis (*p* = 0.312) (Table 1).

**Table 1:** Pre- and postdiagnostic anxiety levels of the parents

	S-anxiety scale score		T-anxiety scale score	
	Pre-diagnostic	Post-diagnostic	Pre-diagnostic	Post-diagnostic
n	40	40	40	40
Median (min–max)	42 (20–78)	37 (20–59)	43.5 (21–59)	38.5 (20–64)
Mean ± SD	42.725 ± 14.137	37.675 ± 11.550	42.225 ± 10.149	40.125 ± 11.353
*p* value	0.049		0.321	

When parents' state and trait anxiety scores were compared, the state anxiety levels of the mothers were significantly higher than those of the fathers before the diagnosis according to the Mann–Whitney U test (*p* = 0.02). However, there were no statistically significant differences between the parents in the rest of the anxiety scale scores (*p* > 0.05).

After the diagnosis of their child, the state anxiety levels of the mothers decreased significantly according to the Wilcoxon Sign Test (p = 0.021). However, there was no statistically significant decrease in the trait anxiety levels of the mothers (*p* = 0.962). Likewise, there were no statistically significant decreases in the fathers' state or trait anxiety levels after they learned their child's diagnosis (*p* = 0.711; *p* = 0.221, respectively) (Table 2). Comparing the decrease in the state-trait anxiety levels between the mothers and fathers based on the Mann–Whitney U test, no statistically significant differences were found (*p* = 0.072; *p* = 0.383, respectively).

**Table 2:** Pre- and postdiagnostic anxiety level differences in mothers and fathers

Mother 1, Father 2		Pre-diagnostic state scale	Post-diagnostic state scale	Pre-diagnostic trait scale	Post-diagnostic trait scale
1	n	20	20	20	20
	Median (min–max)	45 (26–69)	39 (22–56)	45.5 (29–59)	45 (24–61)
	Mean ± Std. deviation	47.35 ± 12.193	38.5 ± 12.159	44.15 ± 9.016	42.95 ± 10.709
	*p* value	0.021		0.0962	
2	n	20	20	20	20
	Median (min–max)	34 (20–78)	34 (20–59)	38.5 (21–57)	36 (20–64)
	Mean ± Std. deviation	38.1 ± 14.714	36.85	40.30 ± 11.060	37.30 ± 11.535
	*p* value	0.711		0.221	

The results of comparing sociodemographic data showed that the decrease in state scale anxiety levels was statistically more prominent in unemployed parents than in employed parents according to the Mann–Whitney U test (*p* = 0.02) (Table 3). However, there were no statistically significant differences in the rest of the parameters according to the Mann–Whitney U test (parental status, age, occupation, educational status, income level, marital status, and psychiatric pharmacological treatment (*p* > 0.05) (Table 4).

**Table 3:** Anxiety levels decrease based on working status (n = 40)

Work status	State scale score decrease	Trait scale score decrease
*Employed*		
n	23	23
Median (min–max)	0.00 ((− 25)–16)	0.00 ((− 35)–11)
Mean ± SD	− 0.39 ± 10.53	− 1.70 ± 9.89
*Unemployed*		
n	17	17
Median (min–max)	− 5((− 42)− 13)	− 1((− 30)− 6)
Mean ± SD	− 11.00 ± 15.02	− 2.65 ± 8.19
*p* value	0.02	0.71

**Table 4:** Sociodemographic profile of the parents (n = 40)

Socio-demographic information	Frequency	Percentage
*Age (years)*		
20–29	4	10
30–39	20	50
40–49	13	32.5
50–59	3	7.5
*Parental status*		
Mother	20	50
Father	20	50
*Number of children*		
1–2	27	67.5
3–4	11	27.5
5 and > 5	2	5
*Marital status*		
Married	40	100
Divorced	0	0
*Educational level*		
Primary school	17	42.5
Secondary school	4	10
Highschool	10	25
Undergraduate	8	20
Postgraduate	1	2.5
*Work status*		
Employed	23	57.5
Unemployed	17	42.5
*Household income*		
Low	9	22.5
Intermediate	31	77.5
High	0	0
*Reported psychiatric disease*		
Anxiey releated	2	5
Depression	6	15
None	32	80
*Psychiatric pharmacological treatment*		
Yes	8	20
No	32	80

## Discussion

This study found that the state anxiety level of parents decreased significantly after the diagnosis. This significant result indicates that further explanation of the disease appears to be key to alleviating parental anxiety. Awareness of anxiety, the identification of its causes, and taking preventive measures are crucial for mental health. Despite this, the long-term anxiety levels of the parents did not decrease significantly after the diagnosis. The long time spent diagnosing their child with a rare genetic disease and the expected difficulties may affect the trait anxiety level of parents. Effectively conducting newborn screening programs and raising awareness of these diseases with special training among health professionals will enable physicians to be more successful in the diagnosis and lessen the diagnosis time, which can help lessen parental anxiety. Additionally, providing psychological support to the family from the moment they arrive at the hospital can help reduce parental anxiety during the diagnostic process.

Parents who experience severe anxiety after their child’s diagnosis with a rare genetic disease may have difficulties understanding and accepting their child's condition, interpreting events realistically, making appropriate decisions, and using appropriate coping strategies. Parents could have difficulty participating in childcare [5, 35–37]. Inadequate parental attention and health care utilization cause low child adherence to therapy. Parents can provide better care to their children if their stress levels decrease. Additionally, it was found that a low level of parental stress has a protective role against distress in their child. Child adherence to therapy is enhanced by having low stress levels and receiving better care from their parents [27, 38, 39]. Moreover, recent studies have found that decreasing parents' stress levels may improve children's symptoms from chronic disease [28, 29, 40]. These improvements reveal that treatment is a process that requires the participation of the family as well as the treatment team. We believe that early psychological intervention for the parents of a child with a rare genetic disease will contribute positively to their child's treatment process.

The importance of psychological support for the family of a chronically ill child has been underlined in studies due to the high stress, depression, and anxiety levels of parents. For the treatment of emotional distress in chronically sick children and their parents, cognitive-behavioral and existential psychotherapy are commonly used. In this context, it is expected that anxiety levels are high in the parents of children with a rare genetic disease and that psychological support is also required for this group of parents. Cognitive-behavioral and existential psychotherapy can be used to treat emotional distress in parents. This psychotherapy teaches parents how to cope with anxiety and depression. Parents gain the skills to continue their daily lives during the diagnostic process of their child [41].

A previous study found that working parents with a chronically ill child might have persistent or recurrent grief-related feelings, such as shock, anger, confusion, sadness, fear, guilt, and denial [42]. Another study among parents of children with rare diseases showed that full-time working parents have high-stress levels, and they may benefit from the provision of rehabilitation services [18]. In this study, the decrease in state anxiety levels was statistically more prominent in unemployed parents than in employed parents. The slight decrease in the anxiety level in working families might be because the working families do not have ample time to cope with this anxiety. They may have feelings of grief for their child's health and might feel guilty about not spending enough time with their child. Additionally, working parents might be aware of the problems that may be encountered in the future, owing to their wider social environment.

## Limitations

Research to evaluate anxiety levels in the family was conducted with quantitative methods until the 1990s [43]. In this study, the anxiety levels of parents were evaluated by the quantitative method STAI-TX form before and after their child was diagnosed with a rare disease. However, in recent years, the importance and interest given to qualitative research have increased. Accordingly, it is recommended that qualitative and quantitative methods be used in further studies to obtain results that reflect the truth in all conditions.

The STAI form is a reliable personal inventory to evaluate anxiety [31, 44]. However, parents also should be tested for depression because of the common coexistence of anxiety and depression. For example, HADS would be a reliable questionnaire to assess anxiety and depression symptom severity in the parents of ill children [45, 46]. The Psychosocial Assessment Tool (PAT) is another successful example that can be used to determine the family's psychosocial status [47]. For further studies, various tests can be used together with the STAI form to assess parents' requirements for psychological support.

Previous studies have shown that mothers were more psychologically affected than fathers by their child's condition. They experience more stress and pressure than fathers in caring for their ill children [10, 48]. Additionally, a study among parents of children with a rare disability showed that mothers have more parental stress than fathers [42]. In this study, the pre- and postdiagnosis anxiety levels of the mother and father were evaluated. Before the diagnosis, the state anxiety levels of mothers were significantly higher than those of fathers. These findings are compatible with the literature. However, in this study, the pre- and postdiagnostic long-term anxiety levels of parents were not significantly different. The limitation of our study was the sample size. To better understand the differences in anxiety levels between mothers and fathers, the sample size of the study should be increased.

It was observed that the decrease in the state anxiety level in mothers was statistically significant, while the decrease in the state anxiety level in fathers was not statistically significant. It is known that women have a higher risk of depression than men. Additionally, being a mother is a negative factor in parental anxiety. However, women use more adaptive coping techniques than men. Unfortunately, limited data exists on this topic. Future research can be more gender-based to understand how gender affects coping strategies [49, 50].

It was found that mothers of a child with a disability had higher depression levels than mothers with nondisabled children [51, 52]. Similar situations might be encountered in mothers who have a child with a rare genetic disease. In this study, most of the mothers had higher anxiety levels than the average score range. However, there was no control group for mothers who had a child with a rare genetic disease. Additionally, the depression levels of the mothers were not evaluated in this study. These may be the weak points of the study. Researchers can learn more about the psychological effects of being a mother of a child with a rare genetic disease if they include mothers of children with non-rare diseases as a control group in the study and use a depression test during the interviews.

In addition to waiting for a diagnosis, the time after the diagnosis can be challenging for parents. Raising a child with a rare genetic disorder poses obstacles to the freedom of parents and their time to socialize. It was also recorded that much of a couple's time and energy was spent caring for the child. It takes more hours to care for a child with a rare genetic disorder than a healthy child because of the additional care requirements, such as hospital visits and home care. As a result, couples did not spend time together to connect as they had before, which may result in damage to their relationship [13, 42]. Within this frame of reference, the risk of divorce is expected in a couple with a child with a rare genetic disorder. However, there are not enough studies to deeply understand and confirm that statement. In this study, the mother or father of the child was randomly selected. Future studies should evaluate the anxiety level of both parents of the child, which would lead to a better understanding of whether the parents' relationship is affected by having a child with a rare genetic disorder.

The first interview was conducted with parents on the same day of the diagnosis or one day before. To better understand the long diagnostic duration effects on parental anxiety, the first interview should be conducted when parents are first admitted to the clinic. Additionally, the number of pre- and postdiagnosis interviews might be increased. Thus, the effect of the long diagnostic process on the anxiety levels of families could be better understood.

Parents may have a sense of shock and anger during the diagnosis of their child. Such conditions would make it harder to have an interview. Some of the parents might refuse to have any further interviews. Informing parents about the medical process, such as treatment and prognosis, reduces anxiety. During this research, we said that we were there for families with every question and that they could call us whenever they had a problem. Anxious individuals focus more on the negative aspects of the process and see the probability of negativities as much higher than it actually is. They tend to underestimate the internal and external potentials that they can use for coping in the case of negative possibilities. Seeing the real possibilities in this regard and being aware of themselves will help them cope with their anxiety. To achieve this, psychological support is a requirement. Additionally, researchers can include parents of children with non-rare diseases as a control group to understand better the effect of having a rare disease on their mental health.

In this study, the second interviews were conducted in the two weeks after diagnosis. The interviews were suspended until the parents were available. It would be recommended for future studies that parents should be well informed about the study while informed consent is obtained. In addition, families should be given time to accept and recover from the diagnosis before having the second interview.

## Conclusion

This study showed that parents of a child with a rare genetic disease have lower state anxiety levels after learning about their child's diagnosis of the disease. In this context, a good explanation by the professional ream of the situation and the difficulties to be experienced in the future has an important role in reducing the state anxiety levels of the parents. However, there was no significant decrease in trait anxiety levels, representing the long-term anxiety level of parents, after their child was diagnosed. The diagnosis affected the state anxiety levels, not the long-term anxiety levels of the parents. In addition, in line with previous studies, the state anxiety levels of working parents were higher than those of nonworking parents.

These results reveal the necessity of psychological support for parents with genetically rare children, especially working parents and mothers. We believe that early psychological intervention in parental distress and developing knowledge of expected circumstances helps them cope with everyday challenges and reintegrates families into society. More importantly, providing social support to the patient and their parents along with the diagnosis will be beneficial in the treatment process.

## Supplementary Information

**Additional file 1**. **Additional table1 S1:** Type of rare disease. **Additional table1 S2:** Evaluation of anxiety scores of the parents according to the average STAI formanxiety level.

## Data Availability

The datasets used and/or analyzed during the current study are available from the corresponding author on reasonable request.
