# Supplementary material for: Evaluation of the parents’ anxiety levels before and after the diagnosis of their child with a rare genetic disease: the necessity of psychological support
Source: Orphanet J Rare Dis. 2021 Sep 28;16:402. doi: 10.1186/s13023-021-02046-2 (PMC8480067; doi:10.1186/s13023-021-02046-2)
Supplement: Supplementary file 1 — Additional file 1. Additional table1 S1: Type of rare disease. Additional table1 S2: Evaluation of anxiety scores of the parents according to the average STAI formanxiety level. [file 13023_2021_2046_MOESM1_ESM.docx]

**Supplementory Information**

Additional table 1

| Bartter Syndrome |
| --- |
| Cholestasis, Progressive Familial Intrahepatic Type 3 |
| Trichorhinophalangeal Syndrome |
| Dravet Syndrome |
| Hyperphenylalaninemia, BH4-deficient, C |
| Glutathione Synthetase Deficiency |
| Mucolipidosis III alpha/beta |
| Macrocephaly/autism Syndrome Due To PTEN Mutations |
| Neurodevelopmental Disorder With Or Without Hyperkinetic Movements And Seizures Due To *GRIN1 M*utations |
| Mowat-Wilson Syndrome |
| Mucolipidosis III Gamma |
| Tuberous Sclerosis |
| Charcot-Marie-Tooth Disease |
| Zellweger Syndrome Type1 |
| Bethlem Myopathy 1 |
| Short/branched-chain ACYL-CoA Dehydrogenase Deficiency |
| Array Abnormality (Microdeletion) |
| Crigler-Najjar Syndrome |
| Multiple Congenital Anomalies-Hypotonia-Seizures Syndrome |
| Achondroplasia |
| Hyperphenylalaninemia Due To DHPR Deficiency |
| Kabuki Syndrome |
| Friedreich Ataxia |
| Congenital Adrenal Hyperplasia |

Title of data: Type of rare disease.

**Description of data:** Types of rare diseases of our patients which we investigated their parental anxietyl evel.

Additional table 2

| **Mother 1,**  **Father 2**  **Parent 3** | | **Pre-diagnostic State Scale** | **Post-diagnostic State Scale** | **Pre-diagnostic Trait Scale** | **Post-diagnostic Trait Scale** |
| --- | --- | --- | --- | --- | --- |
| 1 | **Score** | **n** | **n** | **n** | **n** |
|  | <36 | 3 | 9 | 5 | 5 |
|  | 36-41 | 4 | 3 | 1 | 3 |
|  | >41 | 13 | 8 | 14 | 12 |
| 2 | **Score** | **n** | **n** | **n** | **n** |
|  | <36 | 11 | 11 | 7 | 10 |
|  | 36-41 | 2 | 2 | 4 | 6 |
|  | >41 | 7 | 7 | 9 | 4 |
| 3 | **Score** | **n** | **n** | **n** | **n** |
|  | <36 | 14 | 20 | 12 | 15 |
|  | 36-41 | 6 | 5 | 5 | 9 |
|  | >41 | 20 | 15 | 23 | 16 |

Title of data: Evaluation of state and trait anxiety scores according to the average

**Description of data:** The average state and trait anxiety scale scores varied from 36-41. Additional table 1 shows how many parents, mothers, and fathers have high, low, or average anxiety levels.
